# Relationship Satisfaction and Body Image-Related Quality of Life as Correlates of Sexual Function During Pregnancy: A Systematic Review

**DOI:** 10.3390/healthcare13233020

**Published:** 2025-11-22

**Authors:** Razvan-Ionut Daniluc, Marius Craina, Alina Andreea Tischer, Andrei-Cristian Bondar, Lavinia Stelea, Mihai Calin Bica, Loredana Stana

**Affiliations:** 1 Doctoral School, Faculty of Medicine, “Victor Babes” University of Medicine and Pharmacy Timisoara, 300041 Timisoara, Romania; razvan.daniluc@umft.ro; 2 Department of Obstetrics and Gynecology, Faculty of Medicine, “Victor Babes” University of Medicine and Pharmacy Timisoara, 300041 Timisoara, Romania; craina.marius@umft.ro (M.C.); stelea.lavinia@umft.ro (L.S.); 3 Department of Otorhinolaryngology, “Victor Babes” University of Medicine and Pharmacy Timisoara, 300041 Timisoara, Romania; 4 Faculty of General Medicine, “Titu Maiorescu” University, Calea Văcărești 187, 040051 Bucuresti, Romania; andrei.bondar@prof.utm.ro; 5 Department I, Discipline of Anatomy and Embryology, Faculty of Medicine, “Victor Babes” University of Medicine and Pharmacy Timisoara, 300041 Timisoara, Romania; loredana.stana@umft.ro

**Keywords:** pregnancy, body image, sexual dysfunction, physiological, marital relations, quality of life

## Abstract

*Background and Objectives:* Sexual function often fluctuates during pregnancy, yet the contributions of body image-related quality of life (BI-QoL)—operationalized via body image instruments such as the Body Exposure during Sexual Activities Questionnaire (BESAQ) and pregnancy-specific body image scales—and relationship satisfaction remain inconsistently quantified. This systematic review aimed to synthesize evidence on the associations between BI-QoL, relationship satisfaction, and female sexual function in pregnant women. *Methods:* Following PRISMA 2020, PubMed/MEDLINE, Scopus, and Web of Science were searched up to 23 August 2025. Eligible studies enrolled pregnant women and reported quantitative data on BI-QoL and/or relationship satisfaction alongside sexual outcomes. Risk-of-bias used design-appropriate tools; findings were narratively synthesized due to heterogeneity. *Results:* Thirteen studies met criteria (predominantly cross-sectional; sample sizes 107–472; one RCT; several couples/longitudinal cohorts). Female Sexual Function Index (FSFI) means clustered in the mid-20s; in a randomized trial, the intervention arm improved FSFI by +1.76 points (22.95 → 24.71; *p* = 0.002). Overall female sexual dysfunction reached 54.7% in the largest cross-sectional sample. Higher body exposure anxiety was associated with ~4.24-fold greater odds of dysfunction across trimesters. Marital satisfaction explained ≈36% of FSFI variance in multivariable models. Pregnancy context factors related to BI-QoL included planned pregnancy (β = −0.273) and third trimester (β = −0.280) indicating better BI-QoL, while more children predicted worse BI-QoL (β = +0.317). In one cohort, BI during sexual activity worsened postpartum versus pregnancy (*p* = 0.01). *Conclusions:* Across diverse settings, poorer BI-QoL and lower relationship satisfaction were consistently linked to reduced sexual function during pregnancy, with desire/arousal most affected. Routine screening and couple-sensitive counseling should be considered as promising, yet still under-tested, strategies that warrant further evaluation in intervention studies.

## 1. Introduction

Pregnancy is a period of rapid somatic, hormonal, and psychosocial change that intersects directly with sexual health, understood as a state of physical, emotional, mental, and social well-being in relation to sexuality rather than mere absence of dysfunction [1]. Contemporary global public-health framing emphasizes sexual health as a positive, rights-based, and life-course domain, which helps situate perinatal intimacy and satisfaction as legitimate care targets rather than ancillary outcomes [2]. Recent conceptual work on sexual well-being further underscores its multidimensional nature—linking pleasure, functioning, and relational satisfaction with broader social and structural conditions—and argues that sexual well-being is central to public health agendas [3]. Within this transition, mood and stress symptoms are common and clinically meaningful; recent syntheses estimate perinatal depression around one quarter worldwide, with symptoms persisting for some parents late into the first postpartum year [3,4,5]. These mood and stress symptoms are plausibly associated with changes in desire, comfort, and closeness [3,4,5].

These changes often converge on body image—the multidimensional appraisal of appearance, function, and feelings toward one’s body. Measurement is evolving: up-to-date overviews catalog pregnancy-specific instruments and caution against relying solely on pre-pregnancy tools that miss gestationally salient concerns (abdominal visibility, skin changes, and perceived functionality) [5,6]. Purpose-built measures like the Body Image in Pregnancy Scale (BIPS) capture these domains and have been adapted across settings [7], while legacy questionnaires such as the Body Shape Questionnaire (BSQ) remain widely used and interpretable where pregnancy-specific tools are unavailable [8]. In parallel, the Body Exposure during Sexual Activities Questionnaire (BESAQ) specifically indexes body image-related quality of life in sexual contexts by capturing anxiety about exposing particular body areas during intimacy; higher BESAQ scores have been linked to greater odds of female sexual dysfunction across pregnancy and to postpartum worsening of body image during sexual activity in perinatal cohorts. Sexual function is commonly operationalized with the Female Sexual Function Index (FSFI), which summarizes desire, arousal, lubrication, orgasm, satisfaction, and pain, and has strong psychometric support across community and clinical samples [9,10]. In parallel, new perinatal body image instruments continue to appear, reflecting context-specific cognitions during pregnancy and early parenthood [11]. Together, these tools allow investigators and clinicians to move from generic descriptions of “sexual changes” toward tractable constructs that can be assessed, trended, and targeted. Across large regional and clinic-based samples, application of the conventional FSFI total cut-off suggests that female sexual dysfunction (FSD) during pregnancy is common, with prevalence estimates ranging from roughly one-third to over three-quarters of participants, and the highest burden often observed in late gestation, highlighting that clinically significant sexual difficulties are not rare, even among otherwise healthy pregnancies [11,12,13].

Because pregnancy can be a dyadic transition for those in relationships, relationship processes (warmth, communication, conflict management, and stress spillover) provide essential context. Longitudinal work consistently indicates that many couples experience modest declines in relationship satisfaction across the transition to parenthood and into the early postpartum, with substantial variability shaped by support and stress exposure—patterns directly relevant to how bodily self-perceptions translate into sexual comfort or avoidance [12,13,14]. This framework positions relationship climate as a plausible moderator of the link between body image concerns and sexual well-being during pregnancy.

Finally, contemporary body image science highlights not only dissatisfaction but also positive dimensions—such as appreciation for what the body can do—which may buffer distress and sustain intimacy even amidst visible change and fluctuating desire [15]. These observations align with contextual, circular models of women’s sexual response—most notably Basson’s model—which emphasize that desire and arousal are shaped by emotional intimacy, cognitive appraisal, and relational context rather than by spontaneous drive alone [13]. Bringing these strands together motivates a background in which sexual function during pregnancy is seen as the product of dynamic interactions among bodily change, cognitive–emotional appraisal, and relational context.

The objective of this systematic review was to synthesize and critically appraise quantitative evidence on how body image-related quality of life and relationship satisfaction are associated with women’s sexual function during pregnancy (FSFI total and domains, FSD), while exploring heterogeneity by trimester, parity, pregnancy planning, mental-health symptoms, culture, and study design, including dyadic data and early postpartum follow-up where available. We hypothesize that poorer BI-QoL and lower relationship satisfaction are independently associated with lower FSFI scores and higher odds of FSD—with the largest effects in desire and arousal—and that a more supportive relationship climate attenuates the negative impact of body image concerns on sexual function.

## 2. Materials and Methods

### 2.1. Protocol and Reporting Framework

We designed the review a priori and reported it in accordance with PRISMA 2020 and PRISMA-S recommendations [16]. The protocol defined the research question, eligibility criteria, information sources, search strategy, screening workflow, data extraction plan, outcomes, and risk-of-bias approach before any records were screened. No amendments were made after screening commenced. Because this project synthesizes published data, ethics approval and informed consent were not required. The review was registered with the Open Science Framework (OSF) with the registration code osf.io/b5v82.

### 2.2. Review Question and PICO

The review asked: among pregnant women, how are body image-related quality of life (BI-QoL) and relationship satisfaction associated with sexual function across pregnancy (and, where applicable, early postpartum)? This research question corresponds to the a priori hypothesis articulated in the Introduction that poorer BI-QoL and lower relationship satisfaction would be independently associated with lower FSFI scores and higher odds of FSD.

The PICO was defined as follows: Population—pregnant women (any trimester), including studies that also followed participants into the early postpartum or that included couples if women’s data were extractable. Interventions/Exposures—body image constructs (BESAQ, BIPS/BIS/BSQ, MBSRQ) and/or relationship satisfaction/quality (e.g., Pregnancy and Relationship Questionnaire marital relationship items (PQMR), marital satisfaction scales, Dyadic Adjustment Scale (DAS)); exposure contrasts could be categorical (planned vs. unplanned pregnancy; high vs. low BI-QoL) or continuous. Comparators—alternative exposure levels within pregnant samples; non-pregnant comparators were allowed only to contextualize effects and were not required. Outcomes (prespecified)—primary: overall sexual function measured by the Female Sexual Function Index (FSFI total) and female sexual dysfunction (FSD) prevalence using author-specified or the conventional cut-off; secondary: FSFI domains, BI-QoL indices (BESAQ total), and relationship-quality scores. Study designs—randomized or quasi-experimental trials, cohort studies (prospective/retrospective), and cross-sectional studies published as full-text peer-reviewed articles.

### 2.3. Eligibility Criteria

We included original human studies that (i) enrolled pregnant women, (ii) measured at least one body image instrument and/or a relationship-quality construct, and (iii) reported at least one sexual function outcome (FSFI total or domains, FSD, sexual satisfaction/frequency) with extractable numeric data (means/SDs, counts/percentages, or effect estimates). In line with this, we use “body image-related quality of life (BI-QoL)” throughout the manuscript as an umbrella term referring to these body image instruments (e.g., BESAQ, BIPS, BSQ, and MBSRQ) that capture dissatisfaction, weight/shape concerns, and body exposure anxiety in sexual situations, rather than to a single standardized BI-QoL scale.

**Figure 1:**
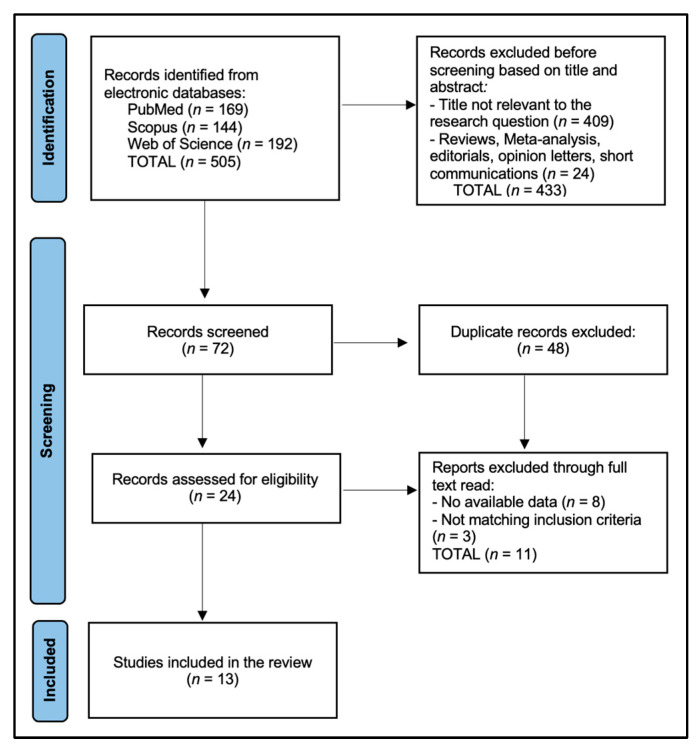
PRISMA flowchart.

Studies restricted to postpartum were eligible only if they also reported pregnancy-period data or longitudinal trajectories anchored in pregnancy. We excluded case reports/series (<10 participants), qualitative-only studies without quantitative outcomes, editorials, letters, conference abstracts without sufficient data, non-human research, and studies in which relevant outcomes were irretrievable after author contact or overlapped with another included cohort (in which case the most complete/least redundant report was retained). For studies contributing relationship satisfaction data, participants were required to be in a current romantic or marital relationship, as the underlying measures (e.g., marital satisfaction scales, dyadic adjustment indices, PQMR) are not applicable to unpartnered individuals. By contrast, studies that assessed body image constructs and sexual function without explicit relationship satisfaction instruments could include both partnered and unpartnered pregnant women. When couples were enrolled, women’s data were extracted primarily; partner data were used for dyadic correlation analyses when reported. PRISMA flowchart is presented in Figure 1.

### 2.4. Information Sources

We searched PubMed/MEDLINE, Scopus, and Web of Science Core Collection from database inception up to 23 August 2025. To enhance completeness, we scanned reference lists of all included articles and key domain reviews and forward-cited included records in Google Scholar.

PubMed/MEDLINE. We combined controlled vocabulary and free-text terms without filters for study design or date. The text string read: (“Pregnancy”[Mesh] OR pregnan* OR antenatal OR prenatal) AND (“Body Image”[Mesh] OR “body image” OR “body image” OR “body dissatisfaction” OR “body image scale” OR BESAQ OR BIPS OR BSQ OR MBSRQ OR “body image self-consciousness” OR “appearance anxiety”) AND (sexual OR “sexual function” OR “female sexual function index” OR FSFI OR “sexual dysfunction” OR dyspareunia OR libido OR desire OR arousal OR orgasm OR lubrication OR “sexual satisfaction”) AND (“Interpersonal Relations”[Mesh] OR “marital satisfaction” OR “relationship satisfaction” OR couple* OR dyadic OR DAS OR PQMR OR “partner relationship”).

Scopus. The TITLE-ABS-KEY query used proximity operators to capture instrument acronyms and full names: TITLE-ABS-KEY(pregnan* OR antenatal OR prenatal) AND TITLE-ABS-KEY(“body image” OR “body image” OR BESAQ OR “Body Image in Pregnancy Scale” OR BIPS OR “Body Shape Questionnaire” OR BSQ OR MBSRQ OR “appearance anxiety” OR “body image self-consciousness”) AND TITLE-ABS-KEY(“female sexual function index” OR FSFI OR “sexual function” OR “sexual dysfunction” OR dyspareunia OR libido OR “sexual satisfaction”) AND TITLE-ABS-KEY(“relationship satisfaction” OR “marital satisfaction” OR “dyadic adjustment” OR DAS OR couple* OR PQMR OR partner).

Web of Science Core Collection. The Topic (TS) query covered title/abstract/keywords across indices: TS = (pregnan* OR antenatal OR prenatal) AND TS = (“body image” OR “body image” OR BESAQ OR BIPS OR “Body Shape Questionnaire” OR BSQ OR MBSRQ OR “appearance anxiety” OR “body image self-consciousness”) AND TS = (“female sexual function index” OR FSFI OR “sexual function” OR “sexual dysfunction” OR dyspareunia OR libido OR “sexual satisfaction”) AND TS = (“relationship satisfaction” OR “marital satisfaction” OR “dyadic adjustment” OR DAS OR couple* OR PQMR OR partner).

### 2.5. Data Items and Extraction

For each study, we captured the following: bibliometrics (first author, year, and country), design, setting, recruitment window, eligibility criteria, sample size (total and analytic), timing relative to pregnancy (trimester[s]) and postpartum follow-up (if any), participant characteristics (age, parity, BMI where available), exposure instruments (e.g., BESAQ, BIPS/BIS/BSQ, MBSRQ; relationship measures—PQMR, marital satisfaction scales, DAS), sexual outcomes (FSFI total and domains; FSD cut-offs; sexual frequency/satisfaction), and covariates included in models. We extracted all available numeric results (means with SD/SE, medians with IQR, counts/percentages, regression coefficients, odds ratios, correlation coefficients, and associated 95% confidence intervals and *p*-values when reported). Where primary studies did not provide confidence intervals, this is indicated in the tables as “95% CI NR” and only the reported *p*-values or point estimates are summarized. When studies reported multiple timepoints, we recorded each distinctly and prespecified pregnancy-period values as primary. For couples’ designs, we extracted women’s outcomes as primary and partner outcomes for dyadic analyses. Two reviewers extracted data independently with cross-checking; discrepancies were reconciled by consensus.

### 2.6. Risk-of-Bias Assessment

Risk-of-bias was appraised independently by two reviewers with design-appropriate tools: Cochrane RoB 2 for randomized trials, the Joanna Briggs Institute (JBI) critical-appraisal checklist for analytical cross-sectional studies, and the Newcastle–Ottawa Scale (NOS) for cohort studies (using the adapted NOS for cross-sectional designs when applicable). Domains covered selection, measurement of exposures and outcomes (including instrument validity and recall period), control for key confounders (age, parity, gestational age, and mental-health symptoms), handling of missing data, and selective reporting.

## 3. Results

Across 13 studies [17,18,19,20,21,22,23,24,25,26,27,28,29], designs and timings were heterogeneous but skewed cross-sectional, with only one randomized trial and several longitudinal cohorts. Sample sizes ranged from N = 107 in a prospective study with 63 complete follow-ups in the U.S. [22] to N = 472 in Turkey [21], with additional large samples such as N = 437 during the COVID-19 period in Iran [24] and a couples design totaling 254 women plus 254 partners in Turkey [26]. Timing covered all trimesters in multiple studies [17,21,24,29], trimester-specific windows (e.g., T2–T3) in Iran (N = 206) [20] and third trimester sampling in Croatia (N = 150) [23], and pregnancy through postpartum in the U.S. (T1 → T3 → 6 months postpartum) [22] and Germany (early → late pregnancy → 8 weeks → 1 year postpartum; N = 136) [27]. Relationship constructs were variously captured via PQMR [17,23], single-item or scale marital satisfaction [24], dyadic adjustment (DAS) in a non-pregnant infertile comparison group [28], and partner-inclusive measures in couples studies [25,26]. Body-image/BI-QoL assessments most commonly included BESAQ [17,22], BIS [26], BSQ [27], and broader instruments such as MBSRQ/BIS [2y] or a Body Image Scale [21], while sexual function was predominantly measured with the FSFI, often complemented by FSD classification and, in couples designs, IIEF or ASEX for partners [19,20,21,24,25,26,28,29], as presented in Table 1.

Relationship climate and BI-QoL indices converged on several consistent signals. In Romania, mean PQMR in pregnancy indicated relatively high comfort/warmth (3.90 ± 1.12) and lower arguing (2.55 ± 1.10) and strain (2.44 ± 1.11), while regression showed better BI-QoL (lower BESAQ) with planned pregnancy (β = −0.273, *p* = 0.007) and third trimester (β = −0.280, *p* = 0.019), but poorer BI-QoL with more children (β = +0.317, *p* = 0.007) [17]. A repeated-measures cohort associated higher body exposure anxiety with ~4.24-fold higher odds of FSD across trimesters [18]. In the Turkish RCT, yoga did not significantly change BESAQ despite FSFI gains [19], whereas postpartum BI during sexual activity worsened versus pregnancy (*p* = 0.01) in a U.S. cohort [22]. Marital satisfaction during the pandemic predicted higher FSFI with a model R^2^ ≈ 35.8%, alongside stress, spouse’s job, income, residence, and gestational age [24]. In Turkey, BIS correlated positively with women’s FSFI yet negatively with partners’ sexual function in a 254-couple sample [26]. Population snapshots highlighted body image burden fluctuations: German data showed BSQ > 100 prevalence of 6.6% (T1), 2.9% (T2), 11.0% (T3), and 10.3% at 1-year postpartum [27]. In infertile women (non-pregnant comparison), higher body image scores co-occurred with higher DAS and FSFI subscales (DAS 113.8 ± 19.73; Body image 308.1 ± 45.8) [28], underscoring contextual differences versus pregnancy samples, as described in Table 2.

FSFI levels clustered in the mid-20s with study- and context-specific variability. In the Turkish RCT, FSFI increased from 22.95 ± 4.14 to 24.71 ± 3.48 in the yoga arm (*p* = 0.002) vs. 24.82 ± 6.15 to 25.79 ± 2.47 in controls (*p* = 0.181), while FSFI–BESAQ correlations were non-significant (r ≈ −0.10; *p* > 0.21) [19]. An Iranian cohort reported FSFI 20.0 ± 8.50 during the pandemic, with inverse correlations to stress (r = −0.203), anxiety (r = −0.166), and depression (r = −0.234), all *p* ≤ 0.001 [24]. Another Iranian study showed modest trimester/BMI shifts: T2 FSFI 25.31 ± 4.61 (normal-weight) vs. 25.25 ± 4.45 (overweight) and T3 23.41 ± 6.61 vs. 25.06 ± 5.00; domain-level disorder prevalences were highest for desire (37.9%) and arousal (37.4%), followed by pain (30.1%), orgasm (18.0%), lubrication (16.5%), and satisfaction (13.6%) [20]. In Turkey, overall FSD prevalence reached 54.7%, with body image associated at OR≈0.98 per unit and additional links to residence, trimester, and parity [21]. Longitudinal U.S. data showed sexual function declined across pregnancy (*p* = 0.017), frequency peaked pre-pregnancy (*p* < 0.0005), and BI during sexual activity worsened postpartum (*p* = 0.01) [22]. A couples study reported women’s FSFI was highest in the second trimester and lowest in the third, while higher women’s BIS was related to higher female FSFI but poorer partner function [26]. A non-pregnant infertile comparison yielded a higher FSFI mean (27.23 ± 3.80), aligning better body image with higher FSFI domains and dyadic adjustment [28], as described in Table 3.

**Figure 2:**
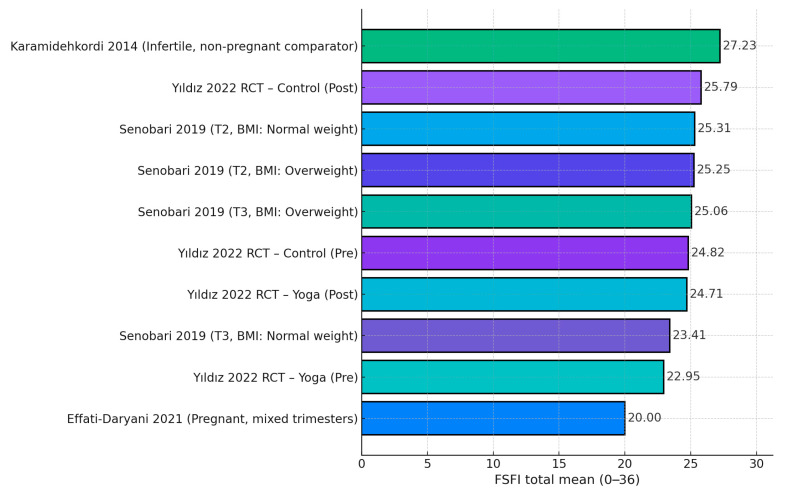
FSFI means across studies [19,20,24,28].

The synthesis showed that mean FSFI scores clustered in the mid-20s but varied by context. The lowest average came from the pandemic-era Iranian cohort (20.00) in Effati-Daryani et al. [24]. In Senobari et al. [20], trimester and BMI shifts were modest: T2 normal-weight (25.31) ≈ T2 overweight (25.25), while T3 normal-weight dipped to 23.41 and T3 overweight was 25.06 (T3 NW vs. T2 NW difference ≈ −1.90 points). In the Turkish RCT, the yoga arm increased from 22.95 to 24.71 (Δ +1.76), whereas the control arm moved from 24.82 to 25.79 (Δ +0.97) [19]. The highest comparator value came from the non-pregnant infertile sample (27.23) in Karamidehkordi & Roudsari [28], as seen in Figure 2.

**Figure 3:**
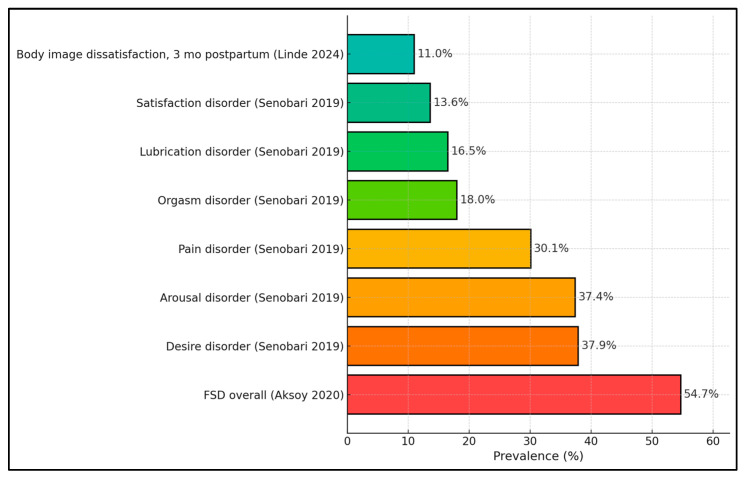
Prevalence of sexual dysfunction across studies [20,21,27].

Aggregated prevalence estimates indicated that overall female sexual dysfunction (FSD) reached 54.7% in the large Turkish sample of Aksoy Derya et al. [21], exceeding any single FSFI domain abnormality. Domain-specific prevalences from Senobari et al. [20] were 37.9% (desire), 37.4% (arousal), 30.1% (pain), 18.0% (orgasm), 16.5% (lubrication), and 13.6% (satisfaction), highlighting desire/arousal as the most frequently affected domains. For body image burden, Linde et al. [27] reported 11.0% prevalence of clinically relevant body image dissatisfaction around three months postpartum (Figure 3).

## 4. Discussion

### 4.1. Summary of Evidence

Across our dataset, FSFI totals clustered in the mid-20s with third-trimester dips and domain-level decrements in desire and arousal—patterns that mirrored several independent cohorts. In a Polish online study (N = 624), total FSFI fell from 28.0 in the first trimester to 25.0 by the third trimester, with desire and arousal most affected [30,31,32]. A UK longitudinal analysis of primiparous women documented a stepwise decline in FSFI medians (27.5 → 24.7 → 21.4, all *p* < 0.0001) and an 86% third-trimester FSD rate using the 26.55 cut-off [33]. Nulliparous women in New York, followed prospectively, also showed lower late pregnancy FSFI and a higher likelihood of scores < 26.55 in the third trimester [31]. Beyond pregnancy, perinatal trajectories from Germany highlighted high inactivity postpartum and persistent risk of FSD (26–35%), with desire most frequently impaired and partnership quality emerging as a relevant context [32]. High cross-sectional FSD prevalence in Turkey (FSFI mean 18.6; FSD 87%) further underscores that trimester-related dips can be substantial in specific settings [34], while Brazilian data suggest adolescents and adults both experience decreased desire and orgasm during gestation, with some postpartum recovery [35].

Our synthesis linked better relationship climate and lower body exposure anxiety to more favorable BI-QoL and sexual outcomes. This comports with a broader literature positioning body image as a moving target during pregnancy and a plausible contributor to sexual well-being. In a multi-trimester review, sexual functioning declined most consistently in the third trimester, and authors cautioned that generic body image tools may miss pregnancy-salient elements (functionality, abdomen visibility, and breast changes) that matter for sexual comfort and self-consciousness [36]. A prospective study of body image dissatisfaction during pregnancy identified psychological factors—self-esteem, pregnancy-specific worries, and sleep quality—over purely anthropometric variables as the strongest correlates of worsening body image profiles [37]. These observations align with our finding that BI-QoL (BESAQ) tracked with contextual factors (e.g., planned pregnancy, and timing), and they support the conceptual model in which pregnancy-specific cognitions about the changing body shape how desire, avoidance, and couple intimacy unfold.

A cross-cutting question is whether BI-QoL or relationship satisfaction showed stronger or more consistent links with sexual function. Across the included studies, relationship satisfaction tended to explain a larger proportion of variance in FSFI scores. In Effati-Daryani et al. [24], marital satisfaction remained a significant predictor of sexual function in generalized linear models, accounting for approximately one-third of the variance (R^2^ ≈ 35.8%) alongside stress and sociodemographic factors. Similarly, in Radoš et al. [24], partnership satisfaction attenuated the contributions of body satisfaction and body image self-consciousness to sexual frequency and satisfaction. In contrast, generic body image instruments (e.g., BSQ, BASS) often showed smaller or non-significant associations with FSFI. The largest and most robust body image effects were observed for BESAQ, which directly indexes anxiety about bodily exposure during sexual activity and was linked to markedly elevated odds of FSD across trimesters and postpartum worsening of body image during sexual activity [18,19,23]. Together, these findings suggest that relationship climate exerts a broad influence on sexual well-being during pregnancy, whereas BI-QoL is particularly impactful when it directly affects comfort with bodily exposure in intimate situations.

Relationship processes likely operate as modifiers rather than mere bystanders. In the German perinatal cohort, lower partnership quality and breastfeeding were independent postpartum risk factors for sexual problems after accounting for mood and obstetric factors [32]. At the same time, relational stressors can directly depress sexual function; for example, a study of young pregnant women in Iran (N = 346) linked intimate partner violence with a two-thirds prevalence of sexual dysfunction and particularly high rates in desire and pain domains, even with a total FSFI mean in the mid-20s [38]. Together with our results—where higher marital comfort and lower strain coincided with more favorable BI-QoL and FSFI—these data reinforce that “dyadic climate” plausibly moderates the strength and direction of body image–sexual function associations across trimesters.

Heterogeneity between countries and cultures in our set (e.g., Romania, Turkey, Iran, the U.S., and Germany) maps onto known cross-cultural differences. In Turkey, very high FSD estimates and concerns about fetal harm, pain, and religious norms were prominent correlates [34], whereas UK and U.S. cohorts emphasized trimester-linked physiologic and psychological changes over sociocultural prohibitions [31,33]. Brazilian work underscored age-specific trajectories (adolescents vs. adults) and postpartum partial rebounds [35]. Across these cohorts, FSFI subdomain analyses consistently pointed to desire and arousal as the domains with the largest decrements or highest rates of dysfunction [31,32,33,34,35,37], mirroring the pattern observed in our included studies, where, for example, desire and arousal were the two most frequently impaired domains (37.9% and 37.4%, respectively) in Senobari et al. [21]. Such variation cautions against rigid application of a universal FSFI cut-off (26.55) to pregnant samples without attention to local norms, instrument language/validation, and sampling frames. In our review, domain-specific patterns (desire/arousal vs. lubrication/orgasm) often shifted with context, suggesting clinicians should prioritize symptom clusters and patient goals rather than case-finding based solely on a single threshold.

Intervention evidence, although still limited, suggests that targeted, couple-sensitive education can mitigate declines in sexual function. A quasi-experimental Iranian study of 123 couples compared group-based sexual education with and without husband participation versus routine care; both intervention arms improved women’s FSFI across follow-ups in the second and third trimesters, supporting sexual health counseling as a feasible antenatal adjunct [39]. Combined with perinatal cohort data showing the salience of partnership quality for postpartum sexuality [32], these findings argue for integrating brief, structured counseling (myth-busting on safety, communication skills, positioning/comfort strategies) within routine antenatal education—complementary to lifestyle and mental-health supports emphasized by body image research [36,37].

Overall, the external literature corroborated our principal signals: (i) sexual function typically decreases across gestation, most steeply in late pregnancy; (ii) desire and arousal are the most sensitive domains; (iii) body image appraisals and relationship climate are mechanistically relevant; and (iv) culturally shaped beliefs and partner dynamics can amplify or buffer dysfunction. Future work should prioritize pregnancy-specific, validated measures of both body image and sexual function; longitudinal dyadic designs with partner-reported outcomes; and randomized, scalable interventions co-targeting body image flexibility, stress regulation, and couple communication—particularly in settings where sociocultural norms heighten avoidance or fear.

Within our dataset, the most robust and interpretable associations between body image constructs and sexual outcomes emerged when body image was assessed with pregnancy- or sexuality-specific instruments such as BESAQ, which directly indexes anxiety about bodily exposure during intimacy. By contrast, generic weight/shape scales (e.g., BSQ, and BASS) showed weaker or more inconsistent links to FSFI outcomes, even in the same samples [21,23,27]. This pattern suggests that instruments capturing gestationally salient concerns and the functional impact of body image on sexual situations are better aligned with the constructs of interest. Accordingly, future work should prioritize pregnancy-specific, validated measures of both body image and sexual function, alongside longitudinal dyadic designs with partner-reported outcomes and randomized, scalable interventions co-targeting body image flexibility, stress regulation, and couple communication—particularly in settings where sociocultural norms heighten avoidance or fear.

### 4.2. Limitations

Evidence was largely cross-sectional and self-reported, limiting causal inference and introducing recall and social-desirability bias. In addition, relationship satisfaction analyses were restricted to women in current relationships, so the findings cannot be generalized to unpartnered pregnant people. Instruments varied (FSFI cut-offs and BI tools), reducing comparability and precluding meta-analysis. Many studies under-reported confounders (mood symptoms, parity, BMI, and comorbidities) and partner outcomes; cultural heterogeneity also constrained generalizability. Some cohorts lacked complete numerical reporting or trimester stratification, and couples’ data were inconsistently available.

## 5. Conclusions

BI-QoL and relationship satisfaction showed robust, directionally consistent associations with sexual function during pregnancy, although the predominantly cross-sectional designs preclude conclusions about causality or direction of influence. The highest dysfunction burden involved desire and arousal in the subset of studies that reported FSFI domains, while marital comfort and lower body exposure anxiety were associated with better outcomes. Given these observational findings and limited interventional evidence, antenatal pathways that combine targeted screening with brief, culturally sensitive, couple-focused counseling should be considered as promising but still under-tested strategies. Future work should prioritize validated pregnancy-specific measures, longitudinal dyadic designs, and randomized interventions to clarify mechanisms, test causality, and identify scalable solutions.

## Figures and Tables

**Table 1:** Background of the included studies.

Study (Year, Country)	Design and N	Timing	Relationship Measure(s)	Body-Image/BI-QoL Measure(s)	Sexual/Other Measures
Daniluc et al., 2024 (Romania) [17]	Cross-sectional; N = 144	All trimesters	PQMR	BESAQ	FSFI
Dăescu et al., 2023 (Romania) [18]	Repeated-measures cohort; N = NR	T1–T3	NR	BESAQ	FSFI, FSD
Yıldız Karaahmet et al., 2022 (Turkey) [19]	RCT; N = 140	Mid-pregnancy	NR	BESAQ	FSFI pre/post
Senobari et al., 2019 (Iran) [20]	Cross-sectional; N = 206	T2–T3	NR	MBSRQ/BIS	FSFI domains
Aksoy Derya et al., 2020 (Turkey) [21]	Cross-sectional; N = 472	All trimesters	NR	Body Image Scale	FSFI, FSD
Pauls et al., 2008 (USA) [22]	Prospective cohort; N = 107 (63 complete)	T1 → T3 → 6 mo pp	Relationship items	BESAQ	FSFI, sexual frequency
Radoš et al., 2014 (Croatia) [23]	Cross-sectional; N = 150	T3	PQMR	BASS/BISC	Sexual frequency/satisfaction
Effati-Daryani et al., 2021 (Iran) [24]	Cross-sectional; N = 437	All trimesters (COVID-19)	Marital satisfaction (single-item/scale)	NR	FSFI, DASS-21
Khalesi et al., 2018 (Iran) [25]	Cross-sectional couples; N = NR	All trimesters	Marital/partner items	NR	FSFI (women), IIEF (men)
Gümüşay et al., 2021 (Turkey) [26]	Cross-sectional couples; N = 254 women + 254 partners	Trimester-stratified	NR	BIS	FSFI; ASEX-Male
Linde et al., 2024 (Germany) [27]	Longitudinal cohort; N = 136	Early → late preg → 8 wk → 1 yr pp	NR	BSQ	BMI trajectory
Karamidehkordi & Roudsari, 2014 (Iran) [28]	Cross-sectional; N = 130 infertile women	Non-pregnant	DAS	Modified Body Image Questionnaire	FSFI
Şolt Kırca & Dağlı, 2023 (Turkey) [29]	Cross-sectional; N = 200	All trimesters; Turkish & Syrian	Sexual attitudes	NR	FSFI

PQMR = Partner/Relationship items; BESAQ = Body Exposure during Sexual Activities Questionnaire; FSFI = Female Sexual Function Index; FSD = Female Sexual Dysfunction; T1–T3 = Trimesters 1–3; pp = postpartum; MBSRQ = Multidimensional Body–Self Relations Questionnaire; BIS = Body Image Scale (contextual) or Body Image (as labeled by study); BISC = Body Image Self-Consciousness; BASS = Body Areas Satisfaction Scale; IIEF = International Index of Erectile Function; ASEX = Arizona Sexual Experiences Scale; BSQ = Body Shape Questionnaire; DAS = Dyadic Adjustment Scale; NR = not reported.

**Table 2:** Relationship satisfaction and body image-related quality of life.

Study	Relationship Satisfaction (Numeric)	Body-Image/BI-QoL (Numeric)	Findings
Daniluc et al., 2024 (Romania) [17]	PQMR item means (planned + unplanned pooled): Comfort/Warmth 3.90 ± 1.12; Unwilling to confide 2.58 ± 1.09; Relationship strain 2.44 ± 1.11; Arguing 2.55 ± 1.10	BESAQ: Planned pregnancy β = −0.273 (*p* = 0.007); 3rd trimester β = −0.280 (*p* = 0.019); # children β = +0.317 (*p* = 0.007) (higher = poorer BI-QoL)	Planned pregnancy and later trimester associated with better BI-QoL; more children worse.
Dăescu et al., 2023 (Romania) [18]	NR	Higher BESAQ increased FSD risk ~4.24-fold	Repeated-measures: rising BI-exposure anxiety linked to FSD odds.
Yıldız Karaahmet et al., 2022 (Turkey) [19]	NR	BESAQ: no significant pre-post change in either arm	RCT yoga—no BI-QoL change despite FSFI gains.
Senobari et al., 2019 (Iran) [20]	NR	MBSRQ/BIS subscales reported; correlations to FSFI domains	Body satisfaction domains measured; select domain–FSFI links reported.
Aksoy Derya et al., 2020 (Turkey) [21]	NR	Body Image Scale associated with FSD (see OR below)	Each unit change ~OR 0.98 for FSD (direction protective with higher body image).
Pauls et al., 2008 (USA) [22]	Relationship items collected; no numeric PQMR	BESAQ worsened postpartum vs. pregnancy (*p* = 0.01)	BI during sexual activity declined postpartum; see sexual function trajectory.
Radoš et al., 2014 (Croatia) [23]	PQMR: body satisfaction and BISC had limited roles after controlling for partnership satisfaction	BASS/BISC used; numerics NR	N = 150; controlled analyses emphasized partnership satisfaction.
Effati-Daryani et al., 2021 (Iran) [24]	Higher marital satisfaction predicted higher FSFI (GLM)	NR	Model R^2^ ≈ 35.8% with marital satisfaction, stress, spouse job, income, residence, gestational age.
Khalesi et al., 2018 (Iran) [25]	Couple relationship items; numerics NR	NR	Couples design; sexual function affected across pregnancy.
Gümüşay et al., 2021 (Turkey) [26]	NR (focus on couple function)	BIS positively correlated with women’s FSFI; negatively with partners’ sexual function	N = 254 pairs; trimester differences described.
Linde et al., 2024 (Germany) [27]	NR	BSQ > 100 prevalence: T1 6.6%; T2 2.9%; T3 11.0%; 1 yr pp 10.3%	Weight/shape concern rises by late pregnancy/postpartum.
Karamidehkordi & Roudsari, 2014 (Iran) [28]	DAS total 113.8 ± 19.73	Body image 308.1 ± 45.8	Infertile women; higher body image associated with higher DAS and FSFI subscales.
Şolt Kırca & Dağlı, 2023 (Turkey) [29]	Sexual attitudes measured; relationship satisfaction NR	NR	Comparative (Turkish vs. Syrian) sexual attitudes with FSFI.

PQMR = Pregnancy and Relationship Questionnaire marital relationship items; BESAQ = Body Exposure during Sexual Activities Questionnaire; FSFI = Female Sexual Function Index; FSD = Female Sexual Dysfunction; BIS = Body Image Scale/Body Image (as used by each study); BSQ = Body Shape Questionnaire; BASS = Body Areas Satisfaction Scale; DAS = Dyadic Adjustment Scale; GLM = Generalized Linear Model; NR = not reported; ns = not significant; # = number.

**Table 3:** Sexual function.

Study	FSFI Total (Mean ± SD)/Domains	FSD/Sexual Outcomes	Modifiers
Daniluc et al., 2024 (Romania) [17]	NR	FSFI used; detailed domain means NR	Predictors of BESAQ: planned pregnancy (β = −0.273), trimester 3 (β = −0.280), # children (β = +0.317). Relationship items reported (see T2).
Dăescu et al., 2023 (Romania) [18]	NR	FSD odds ↑ ~4.24× with higher BESAQ	Repeated measures across trimesters; direction robust.
Yıldız Karaahmet et al., 2022 (Turkey) [19]	Yoga: 22.95 ± 4.14 → 24.71 ± 3.48 (*p* = 0.002). Control: 24.82 ± 6.15 → 25.79 ± 2.47 (*p* = 0.181).	Improved sexual function in yoga group; no BESAQ change	FSFI ↔ BESAQ correlations ns (r ≈ −0.10; *p* > 0.21 both pre/post).
Senobari et al., 2019 (Iran) [20]	T2 FSFI: 25.31 ± 4.61 (NW) vs. 25.25 ± 4.45 (OW); T3: 23.41 ± 6.61 (NW) vs. 25.06 ± 5.00 (OW). Domains (T2): desire ~3.4; lubrication ~4.5; satisfaction ~4.8; etc.	Disorder prevalence: Desire 37.9%, Pain 30.1%, Lubrication 16.5%, Satisfaction 13.6%, Orgasm 18.0%, Arousal 37.4%	Trimester and BMI effects small; domain-specific differences non-significant (*p* > 0.17–0.95 range).
Aksoy Derya et al., 2020 (Turkey) [21]	NR	FSD prevalence 54.7%	Body image OR ≈ 0.98/unit; residence, trimester, parity related to FSD.
Pauls et al., 2008 (USA) [22]	NR totals	Sexual function declined across pregnancy (*p* = 0.017); frequency highest pre-pregnancy (*p* < 0.0005)	BESAQ worsened postpartum (*p* = 0.01).
Radoš et al., 2014 (Croatia) [23]	NR	Sexual satisfaction/frequency predicted more by partnership satisfaction than body image self-consciousness	Third trimester sample (N = 150).
Effati-Daryani et al., 2021 (Iran) [24]	FSFI total 20.0 ± 8.50	Lower FSFI with higher distress	FSFI vs. stress r = −0.203; anxiety r = −0.166; depression r = −0.234 (all *p* ≤ 0.001); GLM predictors explained 35.8% of variance.
Khalesi et al., 2018 (Iran) [25]	NR	Couples’ sexual function affected during pregnancy	Women’s FSFI and partners’ function both change; numerics NR.
Gümüşay et al., 2021 (Turkey) [26]	NR	Women’s FSFI highest in 2nd trimester, lowest in 3rd; partners’ dysfunction more frequent in 3rd	Positive correlation: women’s BIS ↔ female FSFI; negative: women’s BIS ↔ partners’ function.
Linde et al., 2024 (Germany) [27]	NR	Sexual function not measured	BSQ > 100 rises late pregnancy/postpartum.
Karamidehkordi & Roudsari, 2014 (Iran) [28]	FSFI 27.23 ± 3.80	(Infertile women, contextual)	Higher body image associated with higher FSFI domains and higher DAS.
Şolt Kırca & Dağlı, 2023 (Turkey) [29]	NR	FSFI used; comparative attitudes (Turkish vs. Syrian)	Attitudes related to sexual function; numerics NR.

FSFI = Female Sexual Function Index; FSD = Female Sexual Dysfunction; BESAQ = Body Exposure during Sexual Activities Questionnaire; BIS = Body Image Scale/Body Image (as used by each study); BMI = Body-mass index; NW = normal-weight; OW = overweight; OR = odds ratio; ns = not significant; T2/T3 = second/third trimester; NR = not reported; # = number.

## Data Availability

No new data were created or analyzed in this study.
